# Xuetongsu attenuates synovial inflammation in rheumatoid arthritis by inhibiting the IL-23/IL-17/NF-κB inflammatory axis

**DOI:** 10.3389/fphar.2025.1615519

**Published:** 2025-08-26

**Authors:** Yuxin Chen, Yasi Deng, Hao Zheng, Bin Li, Yupei Yang, Juan Huang, Hanwen Yuan, Mengyun Wang, Wei Wang, Huanghe Yu

**Affiliations:** ^1^ TCM and Ethnomedicine Innovation and Development International Laboratory, Innovative Materia Medica Research Institute, School of Pharmacy, Hunan University of Chinese Medicine, Changsha, China; ^2^ Hunan Provincial Key Laboratory of the Research and Development of Novel Pharmaceutical Preparations, School of Pharmacy, Changsha Medical University, Changsha, China

**Keywords:** rheumatoid arthritis, Xuetongsu, synovial inflammation, IL-23, IL-23/IL-17/NF-κB inflammatory signaling axis, RAFLS cell

## Abstract

**Introduction:**

Persistent synovial hyperplasia, a hallmark of rheumatoid arthritis (RA), can lead to joint deformities. During the pathogenesis of RA, the expression of IL-23 promotes Th17 cell proliferation and IL-17 production, which in turn upregulates TNF-α, IL-1β, and RANKL in RA fibroblast-like synovial cells (RAFLS), forming the IL-23/IL-17/NF-κB inflammatory signaling axis, which further exacerbates synovial inflammation and joint destruction. Therefore, inhibiting the IL-23/IL-17/NF-κB inflammatory signaling axis may help alleviate synovial inflammation and could be a promising approach for treating RA. In our previous studies, we found a natural anti-inflammatory active component, Xuetongsu (XTS), which is the active ingredient in the Chinese Tujia ethnomedicine Xuetong, and it has shown significant effects in inhibiting the inflammatory proliferation of RAFLS.

**Methods:**

The RAFLS model and adjuvant-induced arthritis (AIA) animal model were established, and silenced or overexpressed IL-23, and the anti-inflammatory mechanism of XTS was investigated using Western blotting and immunofluorescence. H&E staining was used to evaluate the efficacy of XTS in inhibiting RA synovial inflammatory hyperplasia. The anti-inflammatory and anti-RA bone destruction efficacy of XTS was evaluated by Masson's trichrome staining, Safranin O-Fast Green (SO-FG), Tartrate resistant acid phosphatase (TRAP) staining and radiological analysis. Blood and biochemical indices were used to evaluate the anti-inflammatory efficacy and safety of XTS.

**Results:**

The findings indicated that XTS exerted no notable influence on downstream molecular pathways such as IL-17 and NF-κB in RAFLS cells with silenced IL-23. However, in RAFLS cells with overexpressed IL-23 and in the RA rats model, XTS exhibited a clear inhibitory effect on the downstream factors, which demonstrated a certain dose-dependent relationship. Histopathological staining and radiological analysis showed that XTS could effectively alleviate foot paw swelling and improve synovial inflammatory hyperplasia and bone destruction in AIA rats. Blood analysis revealed that XTS was not only anti-inflammatory, but also improved haematopoiesis and provided hepatic and renal protection.

**Discussion:**

These findings suggest that XTS targets IL-23 to inhibit the IL-23/IL-17/NF-κB axis, offering new insights into RA treatment. This study provides the first evidence that the natural product XTS exerts anti-inflammatory effects in RA by specifically targeting IL-23. Our findings reveal its molecular mechanism and establish a novel paradigm for developing IL-23-targeted RA therapies, advancing traditional medicine modernization.

## Graphical Abstract

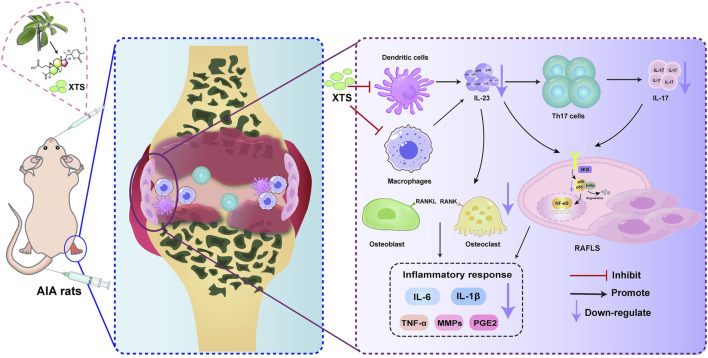

## 1 Introduction

The core pathological feature of rheumatoid arthritis (RA) is persistent synovial inflammation, which causes abnormal proliferation of synovial tissue and progressive bone destruction (Di Matteo et al., 2023; Brown et al., 2024). Clinically manifested as joint swelling and pain, eventually progressing to joint deformity and functional impairment (Chu, 2020; Scherer et al., 2020; Komatsu and Takayanagi, 2022). In this pathological process, the uncontrolled proliferation and excessive activation of rheumatoid arthritis fibroblast-like synovial cells (RAFLS) are the most critical factors leading to synovial inflammation and bone destruction in the joints (Nygaard and Firestein, 2020; José Alcaraz, 2021).

In RA pathogenesis, when RAFLS cells are stimulated by IL-23p19, they increase IL-17 secretion. Subsequently, the released IL-17 activates the NF-κB signaling pathway, stimulating the secretion of various inflammatory mediators (such as TNF-α, IL-6, and IL-1β). (Zaky and El-Nahrery, 2016; Yu et al., 2022). When this inflammatory cascade is amplified, it results in the uncontrolled proliferation and hyperplasia of the synovial tissue (Li and Tsokos, 2021). Clinical studies have consistently demonstrated that RA patients exhibit the levels of IL-23 and IL-17 significantly elevated in both their blood and synovial fluid samples, compared to healthy individuals (Abdo and Tye, 2020). Interestingly, the secretion of IL-23 itself is also positively regulated by these inflammatory mediators, TNF-α and IL-1β (Verstockt et al., 2023). As these factors rise in the RA synovium, it leads to further upregulation of IL-23, perpetuating the self-reinforcing pro-inflammatory feedback loop. In summary, the IL-23/IL-17/NF-κB inflammatory axis is a crucial component of the pro-inflammatory feedback loop, playing a key role in the inflammatory proliferation of the synovium during the pathogenesis of RA. Targeting and inhibiting this inflammatory axis may be a viable approach to interrupt the progression of RA and joint damage (Li and Tsokos, 2021; Najm and McInnes, 2021).

Currently, the clinical treatment of RA primarily relies on four categories of drugs: disease-modifying antirheumatic drugs (DMARDs), nonsteroidal anti-inflammatory drugs (NSAIDs), corticosteroids, and biologic agents. However, traditional DMARDs such as methotrexate (MTX) generally have issues such as slow onset of action, intolerance in some patients, or adverse reactions (Guo et al., 2018); while biologics (such as TNF-α inhibitors) can significantly improve symptoms, they still face limitations such as high infection risks, high costs, the need for long-term injections, and the development of resistance in some patients. While novel oral JAK inhibitors (such as tofacitinib) enhance treatment convenience, they carry the risk of increased thrombosis and infection (Law and Taylor, 2019; Caporali et al., 2024). Notably, recent studies have confirmed that the IL-23/IL-17/NF-κB inflammatory axis is the core mechanism driving the pathological progression of RA. Therefore, the development of safe and effective novel IL-23 inhibitors holds promise for breakthrough solutions in RA treatment.

Xuetongsu, a natural anti-inflammatory triterpenoid compound, has been isolated from this Tujia ethnomedicine plant xuetong (*Kadsura heteroclita* (Roxb.) Craib), which is used to treat rheumatic disease in folk (Wang et al., 2021). Prior studies in our research group have demonstrated that xuetongsu not only exhibits potent anti-inflammatory effects but can also significantly inhibit the proliferation of RAFLS cells. Furthermore, xuetongsu has been shown to suppress collagen-induced arthritis (CIA) mouse paw swelling and bone destruction, suggesting its potential therapeutic value for RA. In terms of the underlying mechanism, the parent plant xuetong can inhibit the expression of the IL-17 factor in the serum of adjuvant-induced arthritis (AIA) rats, thereby suppressing synovial inflammation and hyperplasia. Additionally, xuetongsu has been found to inhibit the phosphorylation of NF-κB in the CIA mice paw tissues, thereby suppressing the inflammatory proliferation of the synovium in the mice’s paws (Yu H. et al., 2019; Zheng et al., 2024). The findings suggest that xuetongsu can regulate key inflammatory proteins in the IL-17/NF-κB signaling axis, significantly improving joint synovial inflammation. IL-23 is a key upstream regulator of the IL-17/NF-κB inflammatory axis. When IL-23 is inhibited, it can block the activation of the IL-23/IL-17/NF-κB signaling axis. This study aims to further investigate whether xuetongsu can act as an inhibitor of IL-23, targeting and suppressing the IL-23/IL-17/NF-κB inflammatory axis to inhibit inflammatory proliferation in the synovium. The IL-23 gene will be silenced or overexpressed to explore the regulatory effects of xuetongsu on the IL-17/NF-κB pathway. Additionally, the inhibitory impact of xuetongsu on the key proteins within the IL-23/IL-17/NF-κB axis will be evaluated in both *in vitro* and *in vivo* models with IL-23 overexpression, and its effects on synovial inflammation in the AIA rat model will be assessed. This research’s findings will clarify xuetongsu’s promising role as a natural, small-molecule IL-23 inhibitor, offering a potential breakthrough in rheumatoid arthritis therapy. This could pave the way for the creation of safer and more effective therapies to combat this challenging autoimmune condition.

## 2 Materials and methods

### 2.1 Reagents

XTS (No. 202006) was extracted from the stem of *Kadsura heteroclite* with a purity of 96.8% (Deng et al., 2024), which is stored at the TCM and Ethnomedicine Innovation & Development International Laboratory. Dulbecco’s modified eagle medium (DMEM), phosphate buffer saline (PBS), and fetal bovine serum (FBS) were sourced from Procell Life Science & Technology Co., Ltd. (Wuhan, China). Protein Phosphatase Inhibitor was obtained from SEVEN Co., Ltd. (Beijing, China); Triptolide (TRI), ethylenediaminetetraacetic acid (EDTA), PMSF, and Bovine type II collagen were obtained from Solarbio Co., Ltd. (Beijing, China); Liquid paraffin were obtained from Sigma-Aldrich Co., Ltd. (MO, United States); BCA protein analysis kits were obtained from Elabscience Biotechnology Co., Ltd. (Wuhan, China); Inactivated *Mycobacterium tuberculosis* H37Ra was obtained from BD Co., Ltd. (NJ, United States); IL-23a adeno-associated virus (AAV) particles were obtained from Jikai Genomics Technology Co., Ltd. (Shanghai, China); IL-23-siRNA was obtained from RiboBio Biotechnology Co., Ltd. (Guangzhou, China); β-actin antibody (AF7018), NF-κB antibody (AF5006), P-NF-κB antibody (AF2006), IL-17 antibody (DF6127), IL-23 antibody (DF13760), and Goat Anti-Rabbit IgG (H+L) HRP (S0001) were obtained from Affinity Co., Ltd. (Jiangsu, China).

### 2.2 Cells

The RAFLS cell was sourced from BeNa Co., Ltd. (Beijing, China). These cells were maintained in DMEM/F12 medium, provided by Procell Life Science & Technology Co., Ltd., and supplemented with 10% FBS and 1% penicillin-streptomycin solution. The cells were incubated at 37 °C with 5% CO_2_ atmosphere.

### 2.3 IL-23-siRNA transfection and its detection assay

RAFLS were inoculated into 6-well plates (2.5 × 10^5^/well) and treated with LPS (400 ng/mL) and 50 nM IL-23 siRNA (without green fluorescence labeling). Following a 24-h incubation period, the protein level of IL-23 was analyzed using Western blotting.

RAFLS were inoculated into 24-well plates (5 × 10^4^/well). Then, they were cultured with LPS (400 ng/mL) and 50 nM IL-23 siRNA (labeled with green fluorescence). After 24 h, the cultured cells were washed three times with PBS buffer. A total of 500 μL of 4% paraformaldehyde was added to each well, and the cells were fixed at room temperature for 10 min to prevent protein degradation and changes in cell structure. After 2–3 washes with PBS buffer to remove residual fixative, cells were permeabilised with 500 μL of 0.1% Triton X-100 per well for 10 min, after which cells were stained with DAPI for 5 min. Finally, the transfection of IL-23-siRNA was evaluated using an OLYMPUS fluorescence microscope (Tokyo, Japan) at ×40 magnification.

### 2.4 Detection test of IL-23-siRNA transfection after drug administration

RAFLS were inoculated in 6-well plates (2.5 × 10^5^/well) and treated with LPS (400 ng/mL) plus 50 nM IL-23-siRNA (without green fluorescence labeling). After 24 h of incubation, the medium was replaced with a solution containing 1% FBS and 1% penicillin/streptomycin, and the treatment group was supplemented with XTS (18 μM). The cells were then cultured for an additional 24 h. Subsequently, cells were harvested and analyzed by Western blotting to determine the expression levels of IL-17 and P-NF-κB.

RAFLS were inoculated into 24-well plates (5 × 10^4^/well). Then, they were cultured with LPS (400 ng/mL), 50 nM IL-23-siRNA (no green fluorescent labeling), and XTS (18 μM). After 24 h, cultured cells were washed three times with PBS buffer. 500 μL of 4% paraformaldehyde was added to each well for 10 min to prevent protein degradation and cell structure changes. After 2–3 washes with PBS buffer to remove residual fixative, cells were permeabilised with 500 μL of 0.1% Triton X-100 per well for 10 min to allow the subsequent antibodies to enter the cells and bind to intracellular antigens. The cells were then blocked with 5% BSA for 30 min to prevent the antibody from binding to non-target proteins and to reduce the background signal, thus improving the specificity. Afterwards, incubate overnight at 4 °C with the diluted primary antibody. The next day, the cells were incubated with fluorescently labelled diluted secondary antibody and protected from light for 2 h. For the sake of labelling the nuclei of the cells to confirm the location and morphology of the cells, the cells were stained with DAPI for 5 min in a light-protected environment, and the plates were sealed with mounting medium that prevents the quenching of fluorescence. Finally, the expression and localisation of P-NF-κB and IL-17 were observed using an OLYMPUS fluorescence microscope (Tokyo, Japan) at ×20 magnification.

### 2.5 IL-23a AAV transfection and its detection assay

RAFLS were inoculated into 6-well plates (2.5 × 10^5^/well) and treated with either LPS (400 ng/mL) or IL-23a AAV particles (MOI = 20). Following a 24-h incubation period, the protein levels of IL-23, IL-17, NF-κB, and P-NF-κB were analyzed using Western blotting.

RAFLS were inoculated into 24-well plates (5 × 10^4^/well) and treated with LPS (400 ng/mL) or IL-23a AAV particles (MOI = 20). Following a 24-h incubation, immunofluorescence was employed to assess the expression and localization of IL-23, IL-17, and P-NF-κB.

### 2.6 Detection test of IL-23a AAV transfection after drug administration

RAFLS were inoculated into 6-well plates (2.5 × 10^5^/well) and treated with LPS (400 ng/mL) alongside IL-23a AAV particles (MOI = 20). Following a 24-h incubation period, the culture medium was replaced with a solution containing 1% FBS and 1% penicillin/streptomycin, supplemented with varying concentrations of XTS (0, 4.5, 9, and 18 μM) or TRI (4.5 μM). The cells were then cultured for an additional 24 h. Afterward, the cells were harvested, and the expressions of IL-23, IL-17, NF-κB, and P-NF-κB were analyzed using Western blotting.

RAFLS were inoculated into 24-well plates (5 × 10^4^/well) and treated with LPS (400 ng/mL) alongside IL-23a AAV particles (MOI = 20). Following a 24-h incubation, immunofluorescence was employed to assess the expression and localization of IL-23, IL-17, and P-NF-κB.

### 2.7 Animals

Male SD rats, weighing between 70 and 90 g, were obtained from Beijing Vital River Laboratory Animal Technology Co., Ltd. These animals were housed in an SPF facility maintained at a constant temperature of 23 °C ± 2 °C. The rats had unrestricted access to both food and water throughout the study. All experimental procedures involving animals were conducted in compliance with ethical standards and were formally approved by the Laboratory Animal Ethics Committee of the Hunan University of Chinese Medicine (permit number: LLBH-202303150006).

### 2.8 AIA+IL-23 rats model induction

In this study, the SD rats were categorized into seven distinct groups: Normal, Model, Model+IL-23, Triptolide (1 mg/kg), and three XTS dosage groups (1 mg/kg, 2 mg/kg, and 4 mg/kg), each consisting of six rats. To induce the AIA model, 100 μL of complete Freund’s adjuvant (CFA) emulsion, containing 400 μg/mL of heat-inactivated Mtb, was administered subcutaneously at the base of the tail to SD rats weighing between 80 and 100 g. Ten days post-injection, with the exception of the Normal and Model groups, the AIA rats received an intra-articular injection of IL-23 adenovirus. These rats were then randomly assigned to the Model+IL-23 group, the Triptolide group, and three different doses of the XTS groups.

### 2.9 Drugs administration and evaluation of the arthritis index

All drugs were prepared by dissolving them in a 0.3% carboxymethyl cellulose sodium (CMC-Na) solution and administered at 0.1 mL per 100 g of rat body weight. The XTS group was given different doses of XTS (1, 2, 4 mg/kg), a triptolide solution of 1.0 mg/kg was given to the positive control group, while the other three groups were administered an equivalent volume of 0.3% CMC-Na solution. Rats in each group were administered orally at the same time point every day for 30 consecutive days. Beginning with the AIA immunization process, the body weight and the thickness of the swollen hind paws in the rats were monitored every 3 days. An electronic scale was used to measure body weight, while a vernier caliper was employed to gauge the thickness of the affected paws. The severity of the swelling was then assessed through a systematic scoring method derived from these measurements.

### 2.10 Histopathological analysis

After a period of 30 days of treatment, after inhalation of 2.5% isoflurane-induced anesthesia in all rats, each rat’s left hind paw was carefully excised using bone scissors and was preserved for a week in 4% paraformaldehyde. A 2-month decalcification procedure employing 10% EDTA came next. Once that was completed, the paw samples were embedded in paraffin and sliced into thin sections. These sections underwent staining with hematoxylin and eosin (H&E), Masson’s trichrome, safranin O-fast green (SO-FG), and tartrate resistant acid phosphatase (TRAP). Ultimately, using a light microscope, the histopathological alterations and their severity were evaluated. The histological lesions in the paws of each rat were categorized on a scale from 0 to 4. A score of 0 indicates that the joint has a normal structure, including joint space, cartilage, bone and synovial tissue, with no signs of inflammation; a score of four indicates marked synovial hyperplasia, the presence of large numbers of inflammatory cells, severe neo-development of bone capillaries, and narrowing of the joint space (Deng et al., 2025).

### 2.11 Radiographic evaluation

Following a 30-day treatment period, after inhalation of 2.5% isoflurane-induced anesthesia in all rats, their right hind paws were carefully excised using bone scissors. These samples were then analyzed using a Micro-CT system (PerkinElmer-Caliper LS Quantum FX Demo, United States) to assess the extent of bone destruction in the hind paws. Using radiographic imaging, the severity of bone damage was systematically evaluated and assigned a score on a scale of 0–4 for each rat. The scoring system classified the damage as follows: 0 represents structurally intact articular bone with no signs of damage, 1 represents mild articular bone destruction, such as small bone defects or bone capsule formation, 2 represents moderate articular bone damage, 3 represents moderately severe damage to the bone, and 4 represents severe articular bone destruction, with significant erosion of articular cartilage and bone (Zheng et al., 2024).

### 2.12 Western blot analysis

Tissue samples from the paws of each group of rats were rapidly frozen in liquid nitrogen, ground to a powder using a tissue grinder, and mixed with RIPA lysis buffer containing protease and phosphatase inhibitors, which was used to extract total protein. Protein concentration was then determined and quantified using a BCA protein assay kit, separated by SDS-PAGE gel electrophoresis, and placed on a PVDF membrane, blocked with skimmed milk for 1.5 h, and then incubated overnight at 4 °C with primary antibody. After washing the membrane four times, the membrane was incubated with the secondary antibody for 1.5 h. Finally, the expression levels of target proteins such as IL-23, IL-17, NF-κB and P-NF-κB were evaluated using a chemiluminescent imaging system.

### 2.13 Blood routine and blood biochemical analysis

Blood specimens were extracted from the orbital venous plexus of rats in each group (n = 6 per group) on the 21st day following the administration of complete Freund’s adjuvant (CFA) for hematological and biochemical analysis. Specifically, 1.0 mL of blood was drawn from each rat and assessed with an automated hematology analyzer. Additionally, blood samples of 2 mL were drawn, then spun in a centrifuge at 1,100 × g for 10 min at 4 °C to isolate the plasma. This plasma was used to assess liver and kidney function, for determining the safety profile of the XTS treatments. During the sacrifice of the animals, arterial blood specimens were obtained from rats’ abdominal aorta in each experimental group (with 6 rats per group) for comprehensive hematological and biochemical analyses. For this step, 1.5 mL of whole blood was collected from each rat and processed using an automated hematology analyzer. Additionally, another 2 mL of blood was drawn, centrifuged under the same conditions, and the resulting plasma was separated to further evaluate liver and kidney function.

### 2.14 Statistical analysis

Data was analysed using SPSS version 22.0 software. All data were expressed as mean ± SEM and were from at least three independent experiments. Normal distribution was satisfied, and Student’s t-test was used for comparison between two groups, and one-way ANOVA was used for comparison between multiple groups. Differences between groups were considered statistically significant when *P* < 0.05.

## 3 Results

### 3.1 XTS targets IL-23 to suppress IL-23/IL-17/NF-κB inflammatory axis

**FIGURE 1:**
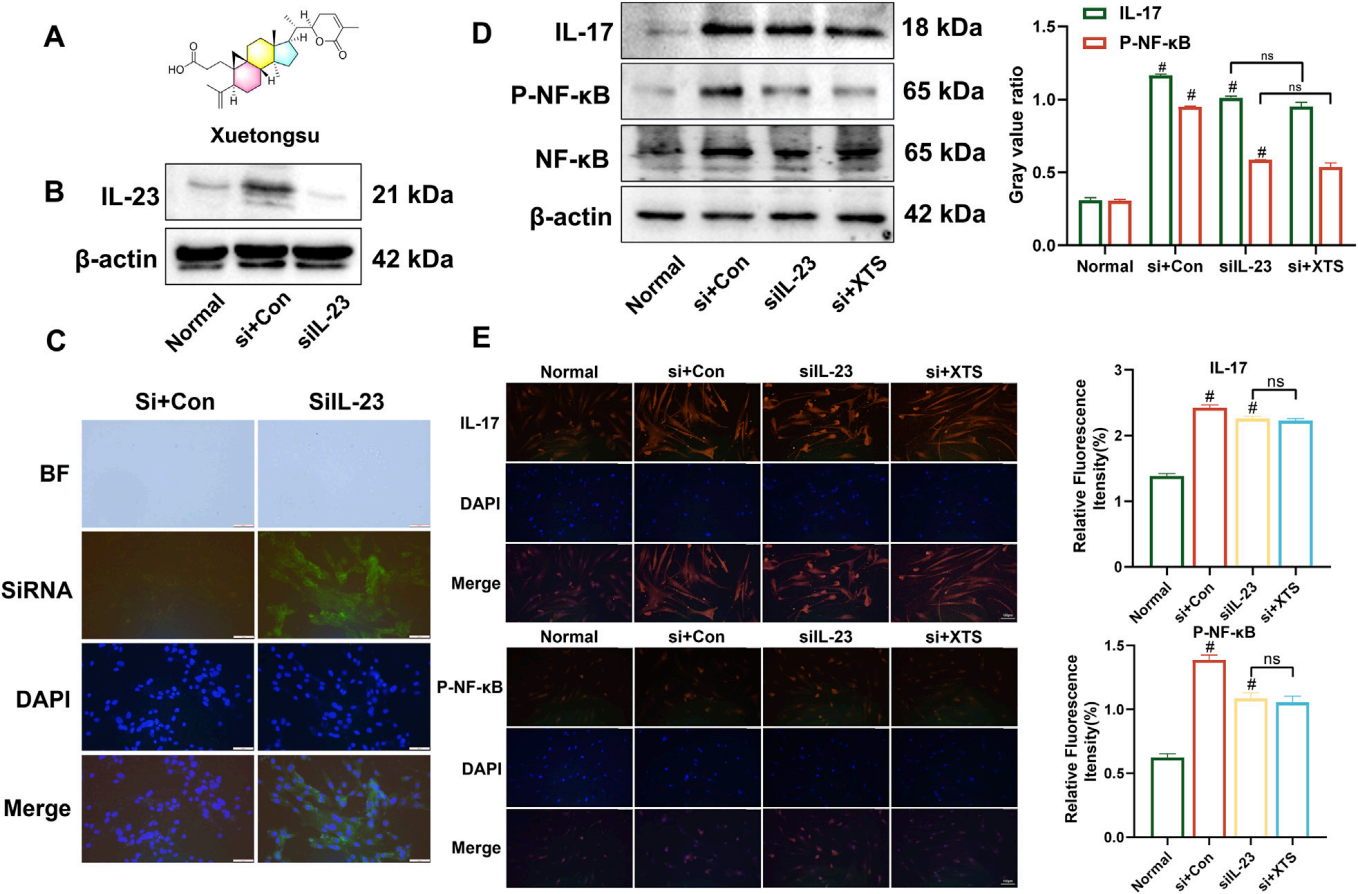
XTS targets IL-23 to inhibit IL-23/IL-17/NF-κB inflammatory axis. **(A)** Chemical structural formula of Xuetongsu. **(B)** The expression of IL-23 after transfected IL-23-siRNA. **(C)** Immunofluorescence analysis was performed using IL-23-siRNA to co-localise with RAFLS cells. Scale bar = 50 μm. **(D)** The expression levels of IL-17, P-NF-κB, and NF-κB proteins in RAFLS cells after transfected with IL-23-siRNA. Values are the mean ± SEM. **(E)** Immunofluorescence image of IL-17 and P-NF-κB in RAFLS cells after transfected IL-23-siRNA. Values are the mean ± SEM. Scale bar = 100 μm. Compared to the normal group, ^*#*^ *P* < 0.05 indicates that the difference is statistically significant. Differences were deemed significant when comparing with the siIL-23 group, where *P < 0.05 and **P < 0.01 were considered noteworthy, *n* = 3.

Xuetongsu (XTS) is a triterpenoid compound of the cycloartan type isolated from the Tujia medicine Xuetong. Its molecular formula is C_30_H_44_O_4_, its molecular weight is 468.67, and its chemical structure is shown in Figure 1A. The Western blot results showed that compared to the normal group or the control group, the expression of IL-23 was significantly reduced in cells treated with IL-23-siRNA (Figure 1B). Immunofluorescence analysis demonstrated that the IL-23-siRNA (green fluorescence labeled) was effectively co-localized with the cells (Figure 1C), indicating that the IL-23 mRNA was successfully silenced in the LPS-induced RAFLS cells, consistent with the above Western blot results. Subsequently, RAFLS cells with silenced IL-23 (siIL-23) were treated with XTS (18 μM). The expression levels of IL-17 and P-NF-κB in the siIL-23 + XTS-treated RAFLS cells were similar to those in the siIL-23-treated RAFLS cells and the control group, showing no significant differences (Figure 1D). This suggests that XTS could not directly downregulate the expression levels of downstream proteins IL-17 and P-NF-κB, which was confirmed by the immunofluorescence results (Figure 1E). These findings indicate that IL-23 may be the key protein targeted by XTS to regulate the IL-23/IL-17/NF-κB signaling axis.

### 3.2 Establishing IL-23 overexpression *in vitro* model by LPS and IL-23 transfection in RAFLS cells

**FIGURE 2:**
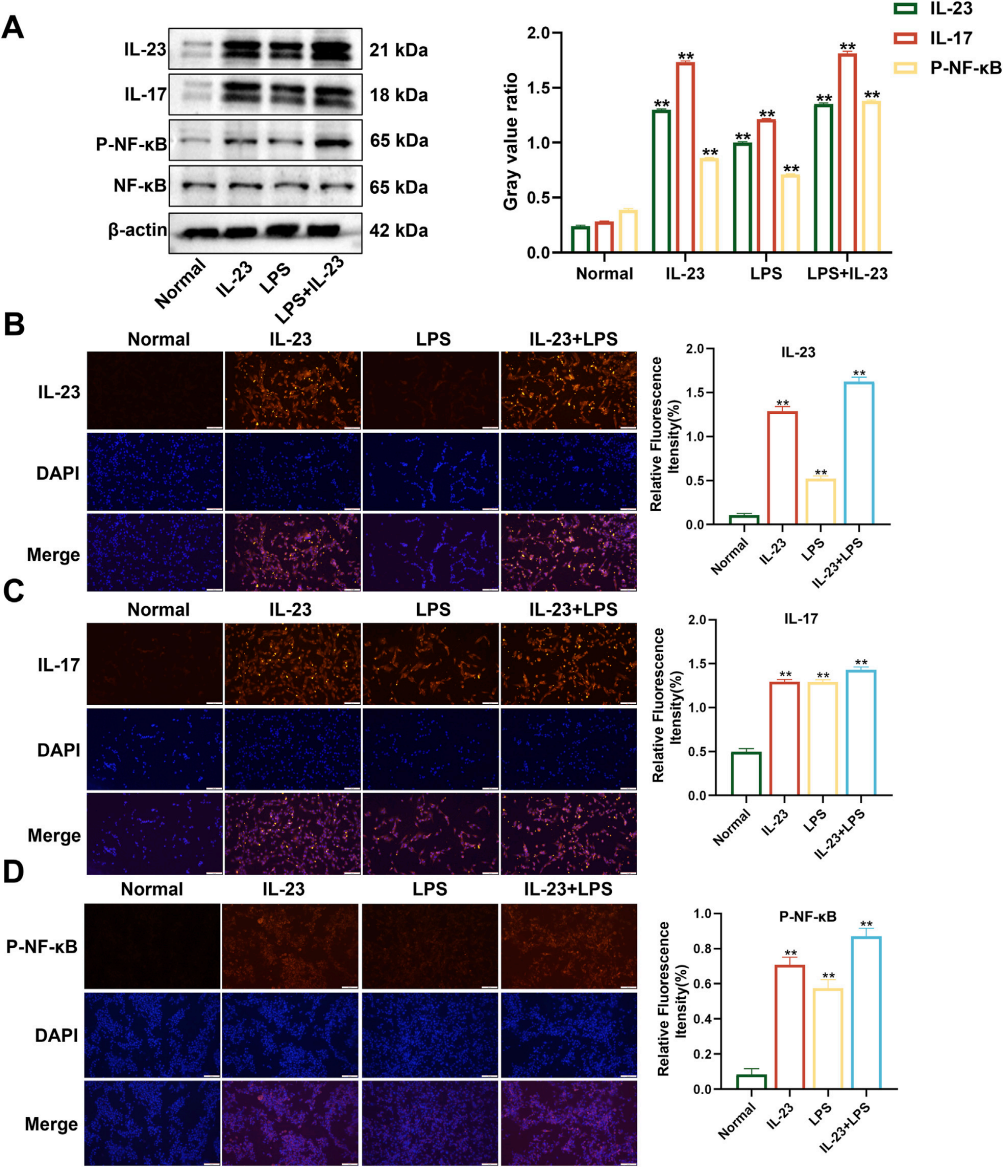
The expression levels of IL-23, IL-17, NF-κB, and P-NF-κB in overexpressing IL-23 RAFLS cells. **(A)** The levels of IL-23, IL-17, P-NF-κB, and NF-κB proteins in overexpressing IL-23 RAFLS cells. Values are the mean ± SEM. **(B–D)** Immunofluorescence images of IL-23, IL-17, and P-NF-κB in overexpressing IL-23 RAFLS cells. Values are the mean ± SEM. Scale bar = 100 μm. Statistical significance was assessed in comparison to the normal group, with *P < 0.05 and **P < 0.01 indicating significance, *n* = 3.

The combination of LPS and IL-23 transfection in RAFLS cells promoted the expression of proteins in the IL-17/NF-κB signaling pathway. RAFLS cells were subjected to three different treatments: transfection with IL-23a AAV (MOI = 20) for 24 h, induction with 400 ng/mL LPS for 24 h, or a combination of LPS and IL-23a AAV transfection for 24 h. Western blotting and immunofluorescence showed that, compared with the normal group, the expression of IL-23, IL-17, and P-NF-κB proteins was significantly upregulated in all three treatment groups (*P* < 0.01) (Figure 2). Specifically, the combined treatment of LPS (400 ng/mL) and IL-23a AAV (MOI = 20) resulted in the highest expression of IL-23, IL-17, NF-κB, and P-NF-κB proteins in RAFLS cells. Therefore, the treatment of RAFLS cells with LPS (400 ng/mL) and IL-23a AAV (MOI = 20) can be utilized to set up an *in vitro* model with IL-23 overexpression.

### 3.3 XTS inhibits the protein expression of the IL-23/IL-17/NF-κB signaling axis in RAFLS cells overexpressing IL-23

**FIGURE 3:**
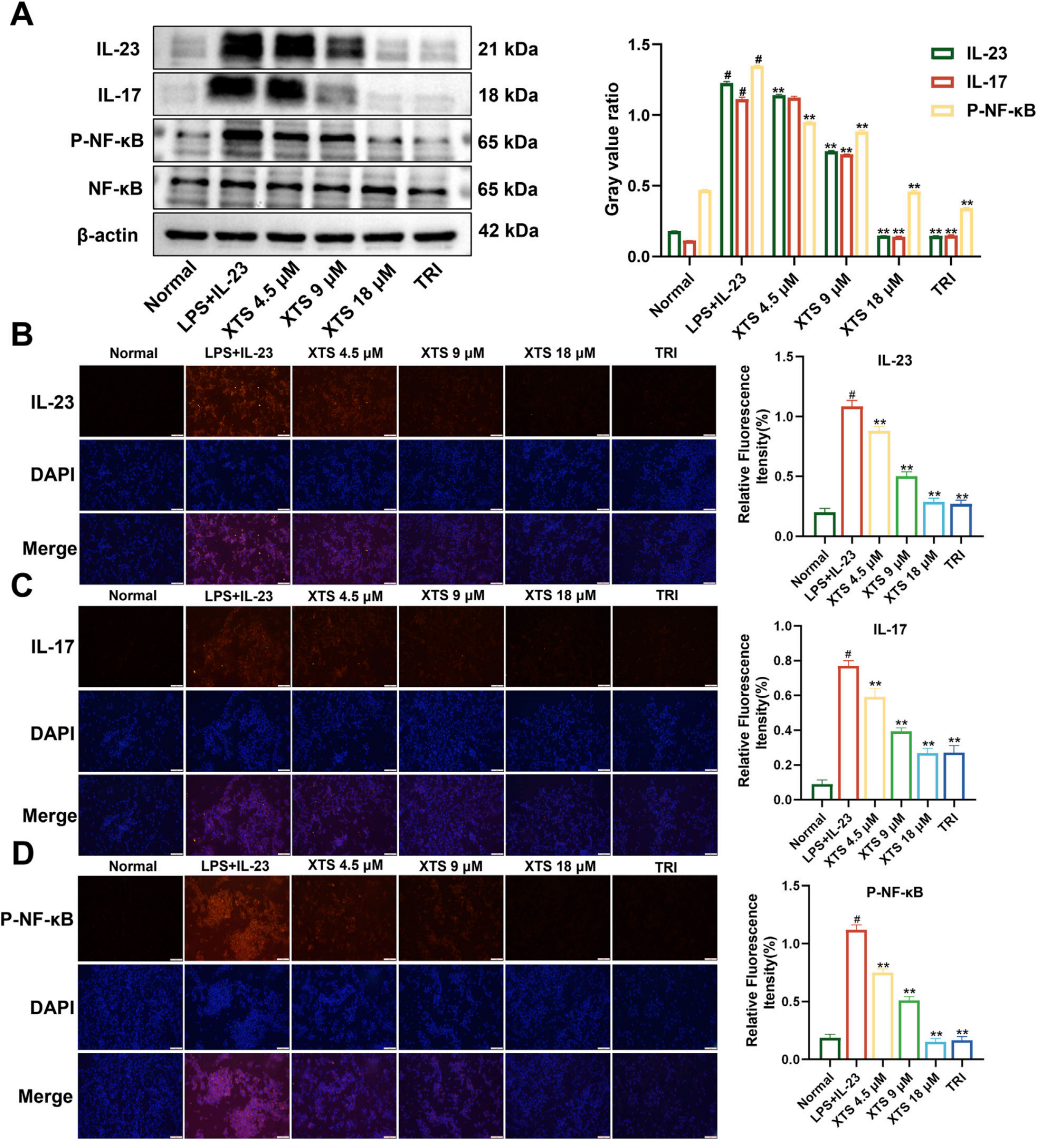
Expression of IL-23, IL-17, NF-κB, and P-NF-κB in IL-23 overexpressing RAFLS cells after XTS treatment. **(A)** The expression levels of IL-23, IL-17, NF-κB, and P-NF-κB proteins in XTS-treated IL-23 overexpressing RAFLS cells. **(B–D)** Immunofluorescence images of IL-23, IL-17, and P-NF-κB in IL-23 overexpressing RAFLS cells treated with XTS. Values are presented as the mean ± SEM. The scale bar = 100 μm. Compared to the normal group, ^*#*^ *P* < 0.05 indicates that the difference is statistically significant. Differences were deemed significant when compared with the LPS+IL-23 group, where *P < 0.05 and **P < 0.01 were considered noteworthy, *n* = 3.

Using the established IL-23 overexpressing RAFLS cell model, the cells were treated with XTS at concentrations of 4.5, 9, and 18 μM. The expression levels of IL-23, IL-17, NF-κB, and P-NF-κB proteins were then assessed through Western blotting and immunofluorescence. Compared to the normal group, the expression of IL-23 and IL-17 was significantly increased in IL-23 overexpressing RAFLS cells, along with increased phosphorylation of NF-κB (Figure 3). Compared with the IL-23 overexpression model group, XTS significantly decreased the expression levels of IL-23 and IL-17 in a dose-dependent manner and inhibited the phosphorylation of NF-κB (*P* < 0.01), indicating that XTS can suppress the IL-23/IL-17/NF-κB inflammatory axis in IL-23 overexpressing RAFLS cells, thereby inhibiting synovial inflammation in RA.

### 3.4 The therapeutic effect of XTS on arthritis in IL-23 overexpressing AIA rats

**FIGURE 4:**
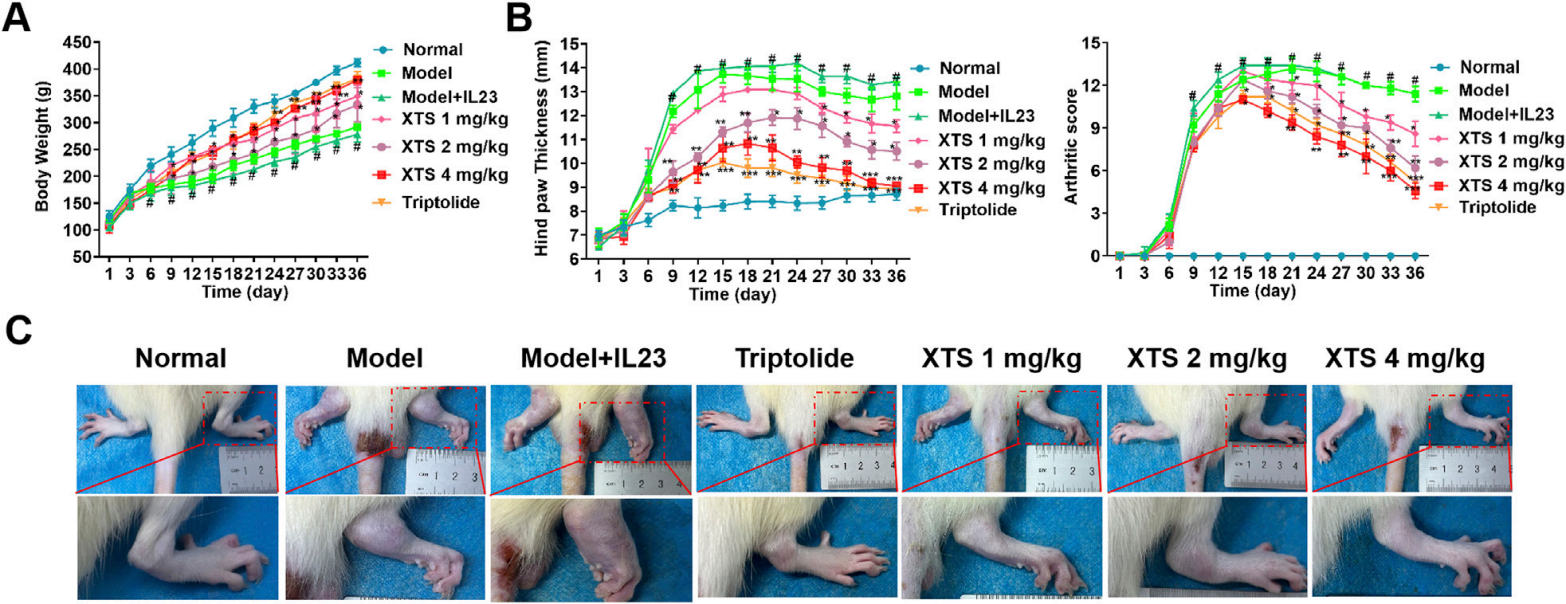
Therapeutic effects of XTS on arthritis progression in IL-23-overexpressing AIA rats. **(A)** Body weight of rats with IL-23 overexpression after treatment with different doses of XTS. **(B)** Quantitative analysis of hindlimb inflammatory markers: The left figure shows the dynamic measurement results of hindlimb thickness in IL-23-overexpressing rats after XTS treatment, and the right figure shows the clinical arthritis score. **(C)** Comparison of hindlimb phenotypes among groups at the end of treatment. All data are expressed as mean ± standard error of the mean (SEM). When comparing the model group with the normal group, ^#^ *P* < 0.05 indicates a statistically significant difference. When comparing the drug administration group with the model + IL-23 group, *P < 0.05 and **P < 0.01 are considered significant, *n* = 6.

After AIA immunization, rats in the model group and the model + IL-23 overexpression group showed significant paw edema and body weight loss, with the IL-23 overexpression group exhibiting more severe paw swelling. Moreover, as shown in Figure 4A, starting on day 3 post-CFA injection, rats in the model and model + IL-23 groups exhibited a notable decrease in body weight relative to the normal group. Notably, XTS treated with 1, 2, and 4 mg/kg dosages protected the AIA rats from weight loss, and their body weight gradually increased after the 9th day of CFA injection. Moreover, XTS markedly reduced the severity of RA. From the 12th day after CFA injection, the AIA rats exhibited a significant rise in hind paw thickness and arthritis index, but XTS significantly reduced paw swelling compared to the model group (*P* < 0.05), indicating its potent anti-inflammatory effect (Figure 4B). Administering XTS orally at doses of 1, 2, and 4 mg/kg led to a significant, dose-dependent reduction in paw swelling in AIA rats (Figure 4C). These results indicate that XTS could enhance the overall health of arthritic rats by mitigating weight gain and related discomfort.

### 3.5 Inhibitory effects of XTS on synovial inflammation in AIA rats with IL-23 overexpression

**FIGURE 5:**
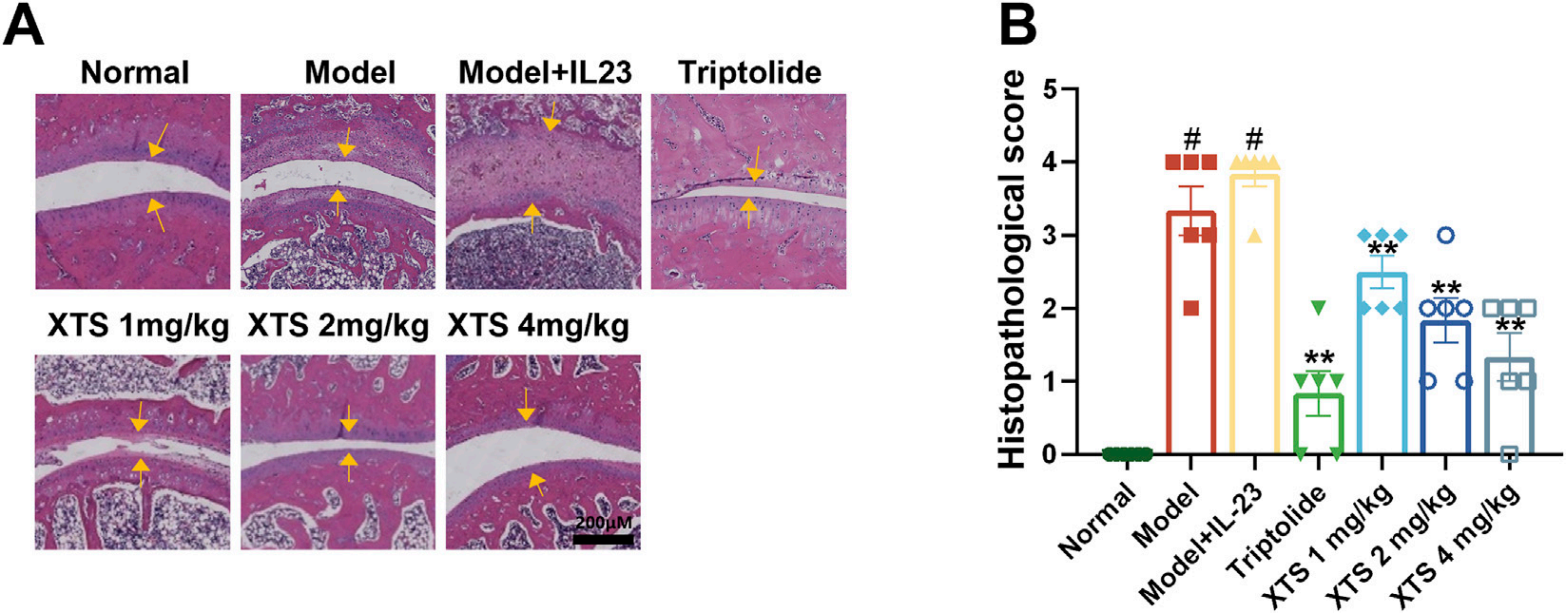
XTS suppresses joint synovial inflammation in IL-23-overexpressing AIA rats. **(A)** The H&E-stained paw sections from IL-23-overexpressing AIA rats administered XTS at different doses. The yellow arrow indicates synovial hyperplasia. The scale bar = 200 μm. **(B)** Histopathological scores for IL-23-overexpressing AIA rats administered varying XTS doses are displayed. Values are expressed as mean ± SEM. When comparing the model group with the normal group, ^*#*^ *P* < 0.05 indicates a statistically significant difference. Compared with the model + IL-23 group, the drug-treated group showed statistically significant differences (*P < 0.05 and **P < 0.01) as determined by statistical analysis, *n* = 6.

As illustrated in Figure 5, H&E staining demonstrated that, relative to the normal group, AIA rats exhibited obvious synovial hyperplasia and joint space narrowing. Furthermore, AIA rats with IL-23 overexpression showed more severe synovial inflammatory hyperplasia and severe joint space narrowing, along with extensive infiltration of inflammatory cells (*P* < 0.05). This suggests that IL-23 can exacerbate synovial inflammation in AIA rats, possibly related to the activation of the IL-17/NF-κB inflammatory axis and the cascade amplification of inflammatory factors due to excessive IL-23 expression. Relative to the model + IL-23 group, XTS administered at doses of 1, 2, and 4 mg/kg could dose-dependently inhibit the synovial inflammatory hyperplasia and inflammatory cell infiltration in AIA rats with IL-23 overexpression (*P* < 0.01), and prevent joint space narrowing, suggesting that XTS has a significant anti-RA effect.

### 3.6 Histopathological observation of the effects of XTS on bone destruction in IL-23 overexpressing AIA rats

**FIGURE 6:**
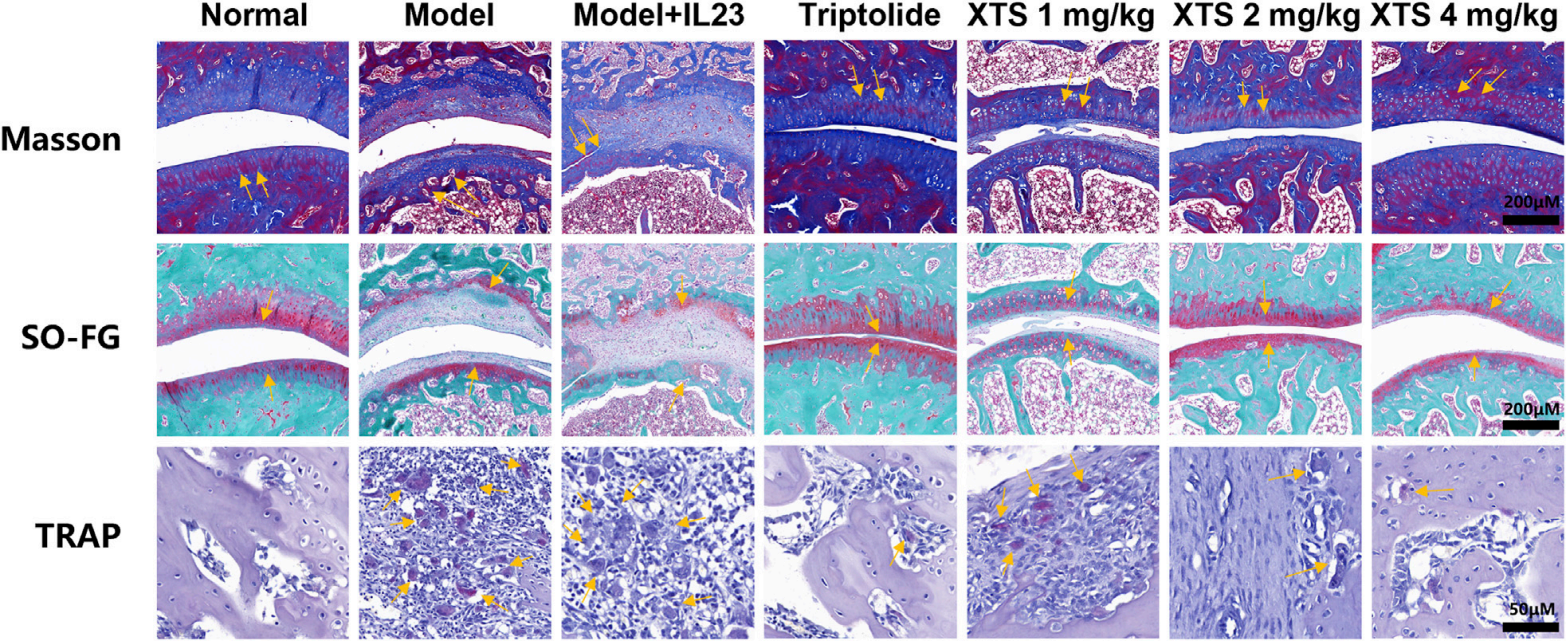
The pathological changes, including Masson staining, SO-FG staining, Scale bar = 200 μm, and TRAP staining, Scale bar = 50 μm in joint tissues of AIA rats.

Histopathological staining of the rat hind ankle joint was performed, including Masson’s trichrome staining, Safranin O-Fast Green (SO-FG), and tartrate resistant acid phosphatase (TRAP) staining as shown in Figure 6. The Masson’s trichrome and SO-FG staining revealed extensive infiltration of inflammatory cells into the ankle joint synovial space and bone marrow space. Cracks were observed in the articular cartilage layer on the ankle joint surface, the quantity of chondrocytes decreased, along with a reduction in matrix staining, indicating obvious inflammatory bone erosion. However, XTS treatment decreased inflammatory cell infiltration in the synovium of the ankle joint and bone marrow space, demonstrating a notable suppression of inflammation in synovial tissue and bone marrow. TRAP staining results indicated a significant presence of osteoclasts in the ankle joints of rats from the model + IL-23 group. In contrast, XTS treatment significantly reduced the number of osteoclasts, indicating that XTS can effectively counter bone destruction by inhibiting the proliferation and differentiation of osteoclasts.

### 3.7 Radiographic observation of the joint in AIA rat

**FIGURE 7:**
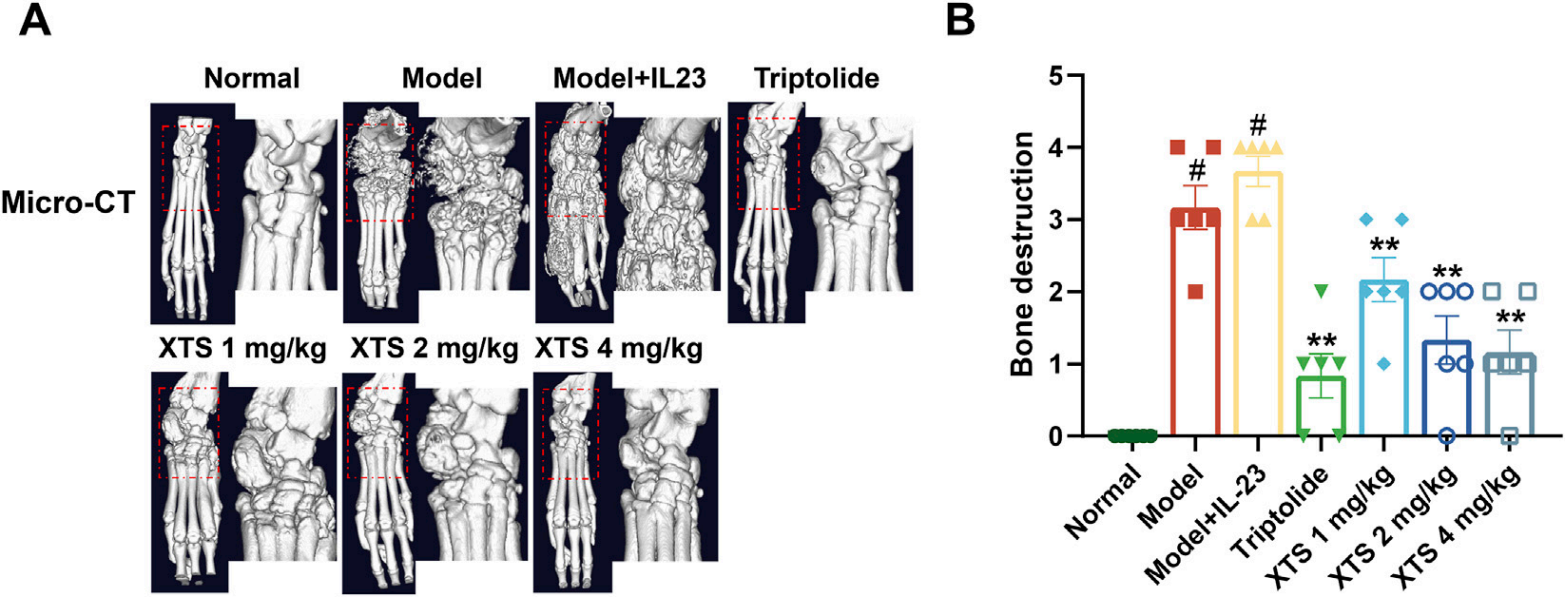
Radiographic assessment of joint bones in AIA rats. **(A)** The micro-CT images of rat paws across all groups. **(B)** Bone destruction in rat paws across all groups. Values are expressed as mean ± SEM. A comparison with the normal group shows that ^*#*^ *P* < 0.05 indicates a statistically significant difference. Significance was determined in comparisons with the model+IL-23 group, where *P < 0.05 and **P < 0.01 were considered noteworthy, *n* = 6.

To further evaluate the effect of XTS on joint bone destruction in arthritic rats, we performed radiological analysis of the rat paw joints using small animal CT. The CT imaging showed that, compared with the normal group, 30 days after adenovirus-mediated IL-23 overexpression, AIA rats exhibited evident erosion of joint cartilage and bone (*P* < 0.05). However, XTS administration at 1, 2, and 4 mg/kg doses provided joint protection and reduced bone destruction (*P* < 0.01) (Figure 7A). These radiological assessments further confirmed that XTS can alleviate the severity of joint synovial inflammation and consequently reduce joint bone destruction.

### 3.8 XTS decreases the protein levels of the IL-23/IL-17/NF-κB inflammatory axis in the paw tissues of AIA rats

**FIGURE 8:**
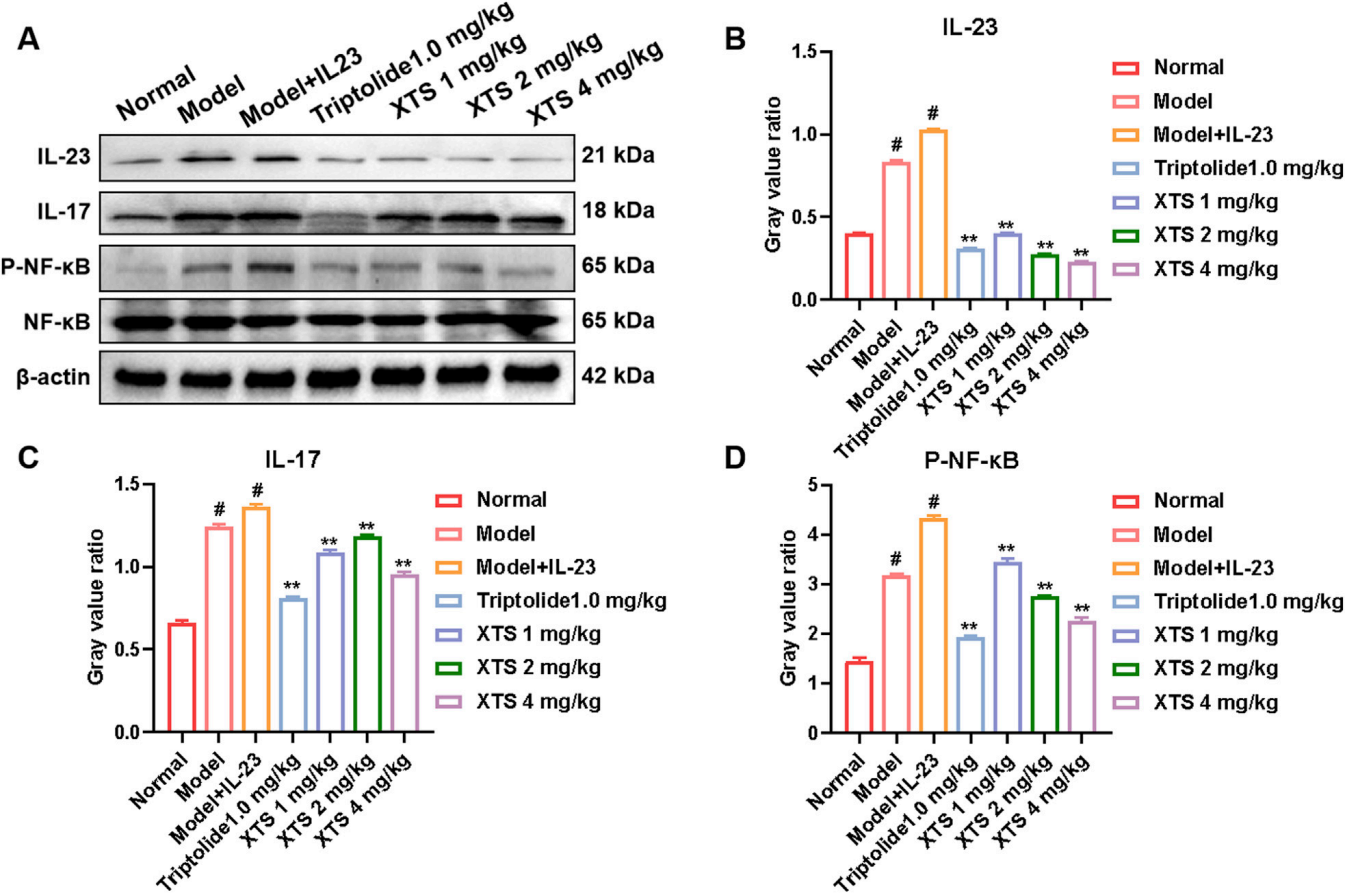
The expression levels of key proteins in the IL-23/IL-17/NF-κB inflammatory axis in the paw tissues of AIA rats. **(A)** The protein levels of IL-23, IL-17, NF-κB, and P-NF-κB in rat paw tissues across all groups. **(B–D)** The gray value of IL-23, IL-17, NF-κB, and P-NF-κB proteins. Values are expressed as mean ± SEM. A comparison with the normal group shows that ^*#*^ *P* < 0.05 indicates a statistically significant difference. Significance was determined in comparisons with the model + IL-23 group, where *P < 0.05 and **P < 0.01 were considered noteworthy, *n* = 6.

In comparison to the normal group, the model group and the IL-23 overexpression group exhibited significantly increased protein levels of IL-23, IL-17, and P-NF-κB in the paw tissues of the rats (*P* < 0.05), demonstrating that IL-23 overexpression activates the IL-23/IL-17/NF-κB inflammatory axis. Importantly, XTS downregulated the expression of IL-23, IL-17, and P-NF-κB in a dose-dependent manner at doses of 1, 2, and 4 mg/kg (*P* < 0.01) (Figure 8), indicating that XTS effectively inhibits the IL-23/IL-17/NF-κB signaling axis in the AIA rat model with IL-23 overexpression.

### 3.9 The effects of XTS on hematological and blood biochemical indices in IL-23 overexpressed AIA rats

**FIGURE 9:**
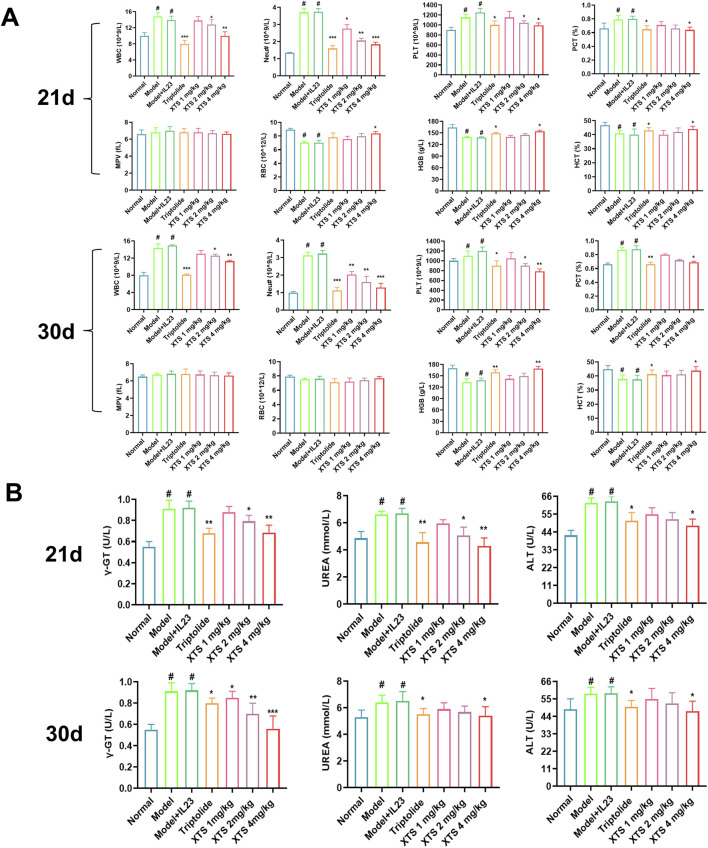
Hematological and blood biochemical indices in XTS-treated overexpressing IL-23 AIA rats. **(A)** Hematological index of the rats after 21 and 30 days of continuous administration. **(B)** Blood biochemical index of the rats after 21 and 30 days of continuous administration. Values are expressed as mean ± SEM. A comparison with the normal group shows that ^*#*^ *P* < 0.05 indicates a statistically significant difference. Differences were deemed significant when compared with the model + IL-23 group, where *P < 0.05, **P < 0.01, and ***P < 0.001 were considered noteworthy, *n* = 6.

After 21 and 30 days of continuous administration, the rats’ hematological and biochemical blood parameters were analyzed, and the results are shown in Figure 9A. Hematological analysis revealed that, in contrast to the normal group, the IL-23 overexpression AIA rats had increased levels of PCT, PLT, Neu#, and WBC, as well as decreased levels of RBC, HGB, and HCT (*P* < 0.05). These results suggested an increased proportion of inflammatory cells in the blood and impaired hematopoietic function, along with an increase in platelet count. However, after 30 days of oral XTS treatment, the proportion of inflammatory cells in the blood of IL-23 overexpression AIA rats decreased, hematopoietic function improved, and platelet count returned to normal levels (*P* < 0.05), indicating that XTS has significant anti-inflammatory and immunomodulatory effects. Blood plasma was collected for biochemical analysis, including assessment of liver function indicators (ALT, γ-GT), and kidney function indicators (UREA). The biochemical analysis of rats’ blood revealed that, in contrast to the normal group, AIA rats exhibited compromised liver and kidney function following immunization and IL-23 induction (*P* < 0.05). Interestingly, XTS treatment can gradually restore liver and kidney function to normal levels in AIA rats (*P* < 0.05) (Figure 9B). Meanwhile, during the 21-day treatment period, both XTS and TRI significantly reduced serum liver function indicators (ALT, γ-GT) in AIA rats. However, when the administration period was extended to 30 days, the γ-GT level in the TRI group rebounded, suggesting time-dependent hepatotoxicity. In contrast, XTS continued to improve liver function and demonstrated good biosafety. In summary, XTS treatment demonstrated good anti-inflammatory capacity and protective effects on liver and kidney function, suggesting its relatively high safety profile.

## 4 Discussion

Synovial inflammatory hyperplasia is a typical pathological feature of RA. Over time, excessive synovial proliferation can lead to joint deformities, which significantly impact patients’ quality of life and work ability, and may also cause psychological distress and social challenges (Sturgeon et al., 2016; Sparks, 2019). As the disease progresses, the incidence of comorbidities such as lung infections increases, and the local inflammation in the synovium and blood vessels becomes more severe (Wang et al., 2019; Radu and Bungau, 2021). This ultimately leads to a higher rate of disability and functional limitations for RA patients. Therefore, it is important to develop innovative drugs to treat RA more effectively.

Xuetong is a herbal medicine of the Tujia ethnomedicine in northwestern Hunan, China. It occupies an important position in the Tujia medical system and is widely used to treat various diseases, especially rheumatic paralysis, bone pain, and RA (Yu H.-H. et al., 2019). XTS is the main triterpenoid active component of xuetong (Cao et al., 2020). Prior research has shown that XTS can suppress the proliferation of RAFLS and has anti-inflammatory properties (Yu H. et al., 2019; Wang et al., 2022). However, the full potential and underlying mechanisms by which XTS suppresses synovial inflammation in RA remain unclear.

Synovial inflammatory hyperplasia is the core pathological process of RA. RAFLS are the primary effector cells driving synovial hyperplasia in RA, primarily distributed in the synovial lining layer and acting as a key cell type implicated in the pathological alterations of RA (Radu and Bungau, 2021). Chronic synovial inflammation can lead to abnormal proliferation of RAFLS, inducing synovial hyperplasia and pannus formation. Additionally, RAFLS can maintain their invasive phenotype, secrete cytokines, and migrate to the cartilage surface, thereby exacerbating synovial inflammation and causing damage to the adjacent bone and cartilage (Filer, 2013; Nygaard and Firestein, 2020). Therefore, the invasively proliferating RAFLS can secrete various proinflammatory cytokines and chemokines to recruit and activate osteoclast precursor cells, further aggravating the inflammatory response. This ultimately leads to the abnormal invasive proliferation of synovial tissue and bone destruction.

IL-23 is a unique heterodimeric cytokine primarily released by activated dendritic cells and macrophages (Verstockt et al., 2023). Research has shown that IL-23 can activate the RANKL to induce Th17 cells, which in turn prompts endothelial cells and synovial fibroblasts to produce inflammatory mediators like IL-17, TNF-α, IL-6, and prostaglandin E2 (PGE2). This cascade of events contributes to the chronic synovial inflammation observed in RA (Lee et al., 2022). Additionally, IL-23 can increase RANK expression in osteoclast precursor cells, stimulating the generation and activation of osteoclasts, accompanied by inflammatory infiltration in the bone and cartilage tissues, ultimately resulting in joint destruction (Bhattacharya et al., 2023; García-Domínguez, 2025). Clinical studies have demonstrated that the IL-23 in the synovium and serum of RA patients is abnormally elevated, and it can serve as a biomarker for RA diagnosis (Lapkina et al., 2025).

IL-17 is a pro-inflammatory cytokine primarily released by Th17 cells, although it can also originate from innate immune cells like NK cells and neutrophils (Adamopoulos and Kuchroo, 2023). Within RA pathogenesis, IL-23 secretion by activated dendritic cells and macrophages initiates a pathogenic cascade wherein cytokine binding to IL-23 receptors on Th17 cells triggers JAK2-mediated phosphorylation of the receptor complex, facilitating STAT docking site formation and nuclear translocation of phosphorylated STAT dimers (Bridgewood et al., 2020); this not only amplifies Th17 proliferation and survival but drives their differentiation into CCR6-expressing pathogenic Th17 subsets (pTh17), which secrete copious IL-17A/F and GM-CSF (Parween et al., 2025). Subsequent IL-17A engagement with receptors on RAFLS and chondrocytes activates the ACT1/TRAF6 complex, phosphorylating IKKβ to degrade IκBα and liberate NF-κB heterodimers (p50/p65) for nuclear translocation, where they transcriptionally upregulate pro-inflammatory cytokines (TNF-α, IL-6, IL-1β), matrix metalloproteinases (MMPs), and RANKL—collectively inducing cartilage degradation and RANKL-mediated osteoclastogenesis (Brembilla and Boehncke, 2023). Crucially, nuclear NF-κB binds the IL-23A promoter to enhance p19 expression, establishing a self-amplifying circuit further potentiated by TNF-α/TNFR1-driven NF-κB hyperactivation and hypoxia-induced HIF-1α upregulation of IL-17R (Ilchovska and Barrow, 2021), thereby forging an “IL-23→Th17→IL-17→NF-κB→IL-23” vicious cycle that perpetuates synovitis, accelerates invasive synovial hyperplasia, and culminates in irreversible bone destruction.

Therefore, the IL-23/IL-17/NF-κB inflammatory axis plays a vital part in RA progression. Our research indicates that silencing the IL-23 gene in RAFLS cells also leads to a reduction in the expression of IL-17 and P-NF-κB. Interestingly, after the IL-23 gene was silenced, treatment with XTS no longer had the ability to further inhibit IL-17 and P-NF-κB levels in these cells. The levels of IL-17 and P-NF-κB expression in RAFLS cells treated with XTS are similar to those in RAFLS cells that have IL-23 silenced on their own. These results indicate that IL-23 could be a key target for XTS intervention in the IL-23/IL-17/NF-κB inflammatory axis. By disrupting the upstream IL-23 signal, the downstream IL-17 and NF-κB-mediated inflammatory responses can be effectively attenuated. This underscores the possibility of focusing on the IL-23 element of this inflammatory pathway as an effective treatment approach for managing RA. Furthermore, to confirm the key role of IL-23 in driving synovial inflammation, we established an RAFLS cell model with IL-23 overexpression. This was achieved by lentiviral transduction of RAFLS cells, followed by LPS stimulation. Compared to LPS induction or IL-23 transduction alone, the combined treatment of LPS (400 ng/mL) and IL-23 lentivirus (MOI = 20) significantly upregulated the levels of IL-23, IL-17, and P-NF-κB proteins in the IL-23 overexpressing RAFLS cells. This aligns with earlier findings that IL-23 can synergize with LPS to amplify the inflammatory cascade, leading to exacerbated synovial inflammation. To further investigate the potential anti-arthritis and bone-protective effects of XTS, an IL-23 overexpression model in AIA rats was established by injecting an IL-23 adenovirus into the joint cavity of AIA rats. In comparison to the AIA rat group, the AIA+IL-23 rat group showed more pronounced synovial hyperplasia, with the joint space largely filled by the proliferating synovial tissue and an increase in the infiltration of inflammatory cells. Micro-CT imaging revealed that 30 days after adenovirus infection, the AIA+IL-23 group exhibited significant bone erosion, along with severe cartilage and bone destruction. XTS at doses of 1, 2, or 4 mg/kg effectively inhibited inflammatory synovial hyperplasia and bone damage in the joints of AIA+IL-23 model rats. Mechanistic studies showed that XTS significantly reduced the expression of IL-23, IL-17, and P-NF-κB proteins in the rats’ paw tissue of the AIA+IL-23 group. These findings suggest that XTS alleviates RA synovial inflammation by modulating the IL-23/IL-17/NF-κB pathway.

Blood analysis revealed that XTS decreased inflammatory cells, WBC, and Neu levels in IL-23-overexpressing AIA rats. XTS also decreased the levels of platelets and other coagulation factors. In contrast, XTS increased the levels of hemoglobin and hematocrit, indicating an improvement in hematopoietic function. Furthermore, XTS exhibited hepatoprotective and nephroprotective effects by reducing the levels of ALT and UREA, which are markers of liver and kidney function, respectively. These results suggest that XTS is an anti-RA drug that can not only suppress synovial hyperplasia but also improve hematopoietic function and provide hepatorenal protection. In summary, the blood analysis data revealed that XTS can reduce the inflammatory cell counts, coagulation factors, and improve the hematological parameters in the IL-23 overexpressing AIA rat model. Additionally, XTS demonstrated hepatoprotective and nephroprotective effects, suggesting its potential as a comprehensive therapeutic agent for RA. It is worth noting that the anti-inflammatory mechanism of XTS in synovial cells exhibits multi-pathway synergistic properties. In addition to the inhibition of the IL-23/IL-17/NF-κB axis confirmed in this study, our previous work has demonstrated that XTS effectively suppresses LPS-induced inflammatory responses in RAFLS and RAW264.7 cells by regulating the JAK2/STAT3 and NF-κB signaling pathways (Deng et al., 2025). Compared with the current mainstream targeted biologics for treating RA, JAK inhibitors can broadly block the JAK-STAT pathway to inhibit inflammatory signals. However, due to the cross-talk between multiple pathways, they are prone to off-target effects, leading to a high incidence of infections and hematological adverse reactions, and the treatment costs are high. IL-17 inhibitors effectively alleviate joint inflammation and bone destruction by specifically neutralizing the pro-inflammatory factor IL-17, but their single-target mechanism limits their ability to regulate the immune microenvironment as a whole; TNF-α inhibitors can rapidly inhibit the key inflammatory mediator TNF-α to improve symptoms, but long-term use may increase the risk of opportunistic infections and malignant tumors due to systemic immune suppression. In contrast, XTS, a naturally occurring small molecule compound targeting IL-23, may exhibit higher selectivity and lower immunogenicity, significantly reducing immunogenicity risks while inhibiting synovial inflammation and demonstrating unique hepatoprotective and nephroprotective functions. Although these characteristics make XTS a promising therapeutic alternative, its development stage inherently has limitations: (1) Human pharmacokinetic data remain limited compared to clinically validated drugs; (2) The optimal dose to balance IL-23 inhibition with organ protection requires further validation; (3) For patients with concomitant autoimmune diseases, the long-term immunomodulatory effects require more comprehensive toxicological assessment.

At the drug development level, this study confirmed that XTS can target and inhibit the IL-23/IL-17/NF-κB inflammatory axis, suppress synovial inflammation and proliferation, and significantly improve paw swelling and inflammatory cell infiltration in an IL-23-overexpressing AIA rat. These findings provide critical preclinical evidence supporting its potential as a candidate drug for RA treatment. Given the current demand for multi-target modulators in the field of RA treatment, XTS holds promise as a complementary therapeutic strategy to existing biologic therapies. In previous studies, we used ultra-high-performance liquid chromatography-MS/MS (UHPLC-MS/MS) and UHPLC-Q-Orbitrap HRMS methods to investigate the metabolism and pharmacokinetics of XTS in rats. The results showed that XTS exhibited a high absolute oral bioavailability of 79.3% in rat models and was primarily excreted via the enterohepatic circulation, laying a solid foundation for the drug development of XTS (Liu et al., 2020). However, the pathological differences between animal models and human disease necessitate further systematic preclinical validation, including validation across different animal models, pharmacokinetic studies, and toxicological research, to comprehensively assess its safety and efficacy. In the future, we will continue to refine related research, elucidate the structure-activity relationships between XTS and key IL-23/IL-17 proteins using surface plasmon resonance (SPR) and molecular docking techniques, conduct long-term toxicity tests and reproductive toxicity screening in accordance with GLP standards, and conduct comprehensive studies on anti-RA effects in other RA animal models or organoid models, with the aim of providing more comprehensive and scientific evidence for the clinical application of XTS.

## 5 Conclusion

In summary, IL-23 has been identified as a key factor in activating the IL-17/NF-κB inflammatory signaling axis and has been confirmed as a key target for XTS to exert its anti-inflammatory effects in synovial inflammation in RA. As an IL-23 inhibitor, XTS reduces the production of IL-17 factors in synovial tissue by targeting IL-23, thereby regulating the IL-23/IL-17/NF-κB inflammatory axis and alleviating synovial inflammation in RA. Crucially, we discovered its ability to improve hematopoietic function and provide liver and kidney protection during RA treatment, challenging the established paradigm of toxicity associated with traditional immunosuppressants, making XTS a pioneering multifunctional therapeutic candidate. These mechanistic breakthroughs provide an actionable foundation for developing next-generation RA therapies that go beyond symptom management to target systemic complications. In the future, our research will utilize AI-driven target analysis technologies to elucidate XTS’s bone-protective pathways and computationally optimize its clinical translation potential, ultimately advancing precision medicine for RA patients.

## Data Availability

The original contributions presented in the study are included in the article/supplementary material, further inquiries can be directed to the corresponding authors.

## Abbreviations

AIA, adjuvant-induced arthritis; CFA, complete Freund’s adjuvant; DMEM, dulbecco’s modified eagle medium; DMSO, dimethyl sulfoxide; EDTA, ethylenediaminetetraacetic acid; FBS, fetal bovine serum; IL-1β, interleukin-1β; IL-6, interleukin-6; IL-17, interleukin-17; IL-23, interleukin-23; LPS, lipopolysaccharide; MMPs, matrix metalloproteinases; NF-κB, nuclear factor kappa B; P-NF-κB, phosphorylated nuclear factor kappa B; PBS, phosphate buffer saline; PGE2, prostaglandin E2; RA, rheumatoid arthritis; RAFLS, rheumatoid arthritis fibrolike synovial cells; RANK, receptor activator of nuclear factor kappa B; RANKL, receptor activator of nuclear factor kappa B ligand; SPF, specific pathogen-free; TRI, triptolide; TNF-α, tumor necrosis factor-ɑ; XTS, xuetongsu.
